# Differential tear metabolomics in blepharokeratoconjunctivitis and herpes simplex keratitis: potential biomarkers for clinical differentiation

**DOI:** 10.3389/fopht.2026.1842136

**Published:** 2026-07-15

**Authors:** Zi-wen Wang, Zhen-ning Wu, Hui-zhen Wang, Shi-you Zhou

**Affiliations:** 1 Department of Ophthalmology, Jiangxi Yingtan People’s Hospital, Yingtan, Jiangxi, China; 2 State Key Laboratory of Ophthalmology, Guangdong Provinical Key Laboratory of Ophthalmology and Visual Science, Zhongshan Ophthalmice Center, Sun Yat-sen University, Guangzhou, China

**Keywords:** blepharokeratoconjunctivitis, clinical differentiation, herpes simplex keratitis, potential biomarkers, tear metabolomics

## Abstract

**Objective:**

To characterize tear metabolomic differences between active blepharokeratoconjunctivitis (BKC) and herpes simplex keratitis (HSK; epithelial type) and identify diagnostic biomarkers.

**Methods:**

Tear samples were collected from 24 HSK patients, 19 BKC patients, and 15 healthy controls from October 2020 to 2021. Diagnoses were confirmed by clinical manifestations, medical history, and nested PCR (nPCR). Metabolomic profiling was performed via LC-MS/MS, and data were analyzed using MetaboAnalyst 5.0.

**Results:**

Compared to healthy controls, HSK exhibited 21 altered metabolites, while BKC showed 19 altered metabolites. After FDR correction, L-isoleucine, L-phenylalanine, pantothenol were significantly expressed lower in both HSK and BKC. Moreover, 4-dodecylbenzenesulfonic acid, carnosol were lower in HSK-specific, and sorbitol was lower in BKC-specific than in control. A combined panel of metabolites 4-dodecylbenzenesulfonic acid, carnosol and sorbitol showed good specificity and sensitivity for differentiation of BKC/HSK. All of them showed significant differences with area under curve values exceeding 0.79.

**Conclusion:**

The disease-specific differential expression of carnosol, 4-dodecylbenzenesulfonic acid in HSK, and sorbitol in BKC provide mechanistic insights into HSV-1 infection and chronic immune-mediated ocular surface inflammation, respectively. The combination of clinical signs with nPCR result and a metabolite panel (4-Dodecylbenzenesulfonic Acid+ Carnosol +Sorbitol) may be optimal for HSK/BKC discrimination.

## Introduction

Blepharokeratoconjunctivitis (BKC) is a chronic ocular surface inflammation involving eyelids, conjunctiva, and cornea, characterized by recurrent lesions (1). Herpes simplex keratitis (HSK), primarily caused by HSV-1, manifests as epithelial, stromal, or endothelial keratitis (2). Both diseases can cause vision impairment upon recurrence due to corneal scarring and neovascularization. Despite their different etiologies, both conditions present with similar clinical manifestations, making differential diagnosis challenging.

Clinical features of BKC and HSK overlap significantly, particularly when manifest as mid-peripheral corneal ulcer and neovascularization, leading to misdiagnosis rates exceeding 30% in children (3). This high misdiagnosis rate stems from the reliance on subjective clinical assessment and the limitations of existing laboratory tests, such as viral culture/PCR, which are constrained by low tear viral load and false negative detection (4). Given the distinct treatment approaches required for each condition, identification of reliable biomarkers for differential diagnosis is essential.

To address this need, tear metabolomics may offer a non-invasive approach to identify disease-specific signatures (5). Tear fluid is a uniquely accessible biofluid that directly mirrors pathological changes at the ocular surface. Metabolomics profiling of tears has demonstrated diagnostic potential across a range of ocular surface diseases, including dry eye disease, glaucoma, and keratoconus (5, 6). Given that BKC and HSK involve distinct inflammatory and infectious pathological mechanisms, we hypothesized that tear metabolomic signatures could effectively differentiate between the two conditions. Therefore, this study aimed to compare tear metabolomic profiles of active BKC and HSK, and screen potential biomarkers for differential diagnosis.

## Methods

### Patients

A total of 24 patients with HSK (epithelial keratitis, HSV-1 nPCR-positive),19 patients with BKC (active blepharitis, HSV-1 nPCR-negative) and 15 healthy volunteers were enrolled in Jiangxi Yingtan People’s Hospital from October 2020 to October 2021. This study was approved and the ethics approval was waived by Jiangxi Yingtan People’s Hospital Ethics Committee (YTH-KY-2020-015). Written informed consent was obtained from all participants.

Inclusion Criteria: i) Presentation with a recurrent, mid-peripheral, grayish-white superficial corneal ulcer with peripheral corneal neovascular vessels. Negative microbial culture results (bacteria and fungi) from corneal scrapings. No history of glaucoma, allergic ocular disease, corneal dystrophy, ocular trauma, or prior ocular surgery. ii) Specific Criteria for HSK Group: Clinical presentation consistent with viral keratitis, often accompanied by a recent history of cold. Positive result for herpes simplex virus type 1 (HSV-1) by nested polymerase chain reaction (nPCR) in tear samples. Iii) Specific Criteria for BKC Group: Clinical evidence of active blepharitis (e.g., lid margin hyperemia, telangiectasia, or meibomian gland dysfunction). Negative result for HSV-1 nPCR in tear samples.

Criteria for Healthy Controls: Age- and sex-matched volunteers with no history of ocular surface disease, systemic autoimmune disease (e.g., rheumatoid arthritis), or recent medication use.

Exclusion Criteria: Individuals were excluded from the study if they met any of the following conditions: i) Systemic diseases: Presence of systemic autoimmune or inflammatory disorders (e.g., ankylosing spondylitis, rheumatoid arthritis), systemic psychiatric disorders, or those currently taking systemic immunosuppressive agents or psychotropic drugs. ii) Drug-induced ocular disease: Corneal ulcers or eyelid margin inflammation secondary to topical medication toxicity or hypersensitivity (e.g., preservative toxicity). iii) Confounding ocular history: History of other ocular surface diseases (e.g., severe dry eye, chemical burns), previous intraocular or corneal surgery, or ocular trauma.

### History collection, routine examination and treatment

The clinical information of all patients were recorded in detail, including age, gender, recurrence or initial onset, course of disease, history of systemic diseases or medications, surgical trauma, and allergies.

Treatment: Patients with epithelial HSK were treated with topical Acyclovir eyedrops (Q.I.D, Guangdong Hengjian Pharmaceutical Inc., Jiangmen, China), Ganciclovir eye gel (Q.N., Hubei Keyi pharmaceutical Inc., Wuhan, China) and oral valacyclovir hydrochloride tablets (0.3 B.I.D, Zhuhai Rundu Pharmaceutical Inc., Zhuhai, China). Patients with active BKC were treated with 0.1% Fluorometholone eyedrops, Pranoprofen Eye Drops, Sodium Hyaluronate Eye Drops (Q.I.D. for all, Santen Pharmaceutical Inc., Tokyo, Japan) and 0.3%Tobramycin & Dexamethasone eye drops (Q.N., TobraDex, Alcon Inc., Texas, USA).

### Tear sample collection for net PCR and metabolomics

Tear samples for metabolomics analysis were collected at the time of initial clinical presentation, prior to the initiation of any topical or systemic treatment, to avoid medication-related confounding of the tear metabolome. The tears were collected with Schirmer’s test strips (Morning Biotechnology Co., Ltd., Guangzhou, China). After 5 minutes insertion into the lateral lower conjunctival sac, the heads of test strips were taken out, trimmed and placed into tubes containing 200 μL viral delivery medium (Zuoke Biotechnology Development Co., Ltd.; Guangzhou, China), centrifuged at 20000 g for 10 minutes. The supernatants were collected and stored at -80 °C until further detection.

### Nested PCR detection

Tear-derived DNA was extracted using the QIAGEN DNeasy Blood & Tissue Kit. Herpes simplex virus type 1 (HSV-1) DNA was detected via nested PCR (nPCR) targeting the glycoprotein D gene. The first-round PCR used outer primers (F: 5′-CTCCATGAGCTTTGTACAAGG-3′, R: 5′-TGCTGATGTACCAGTTGGGG-3′) under 40 cycles (95 °C/15s, 55 °C/15s, 72 °C/20s). The second-round PCR employed inner primers (F: 5′-GGGCTGCTCTGGGAACTACTAC-3′, R: 5′-CCTAGCCCGGCTTGAACAA-3′) with 25 cycles (95 °C/15s, 57 °C/15s, 72 °C/10s) and a 1:10 dilution of the first-round product. Amplification products were visualized on a 2% agarose gel, with a ~250-bp band indicating HSV-1 positivity. This validated protocol detects ≥10 copies/µL HSV-1 DNA, as previously demonstrated (5).

### Tear metabolomics analysis

- Sample Preparation: Tear samples (50 μL) were mixed with 200 μL of ice-cold extraction solvent (methanol:acetonitrile, 1:1 v/v) containing isotope-labeled internal standards. Samples were incubated at −40 °C for 1 hour, followed by centrifugation at 12,000 rpm (13,800 × g, 4 °C) for 15 min.
- Chromatographic Separation: Metabolites were separated using ultra-performance liquid chromatography (UPLC) on a Waters ACQUITY BEH Amide column. The mobile phase consisted of: Phase A: 25 mM ammonium acetate and 25 mM ammonia in water, Phase B: acetonitrile. The column was maintained at 4 °C, with an injection volume of 3 μL.
- Mass Spectrometry: Data acquisition was performed using a Thermo Q Exactive HFX Orbitrap mass spectrometer coupled to the UPLC system. Full-scan MS (resolution: 120,000) and data-dependent MS/MS (resolution: 15,000) were operated in positive/negative switching mode under Xcalibur software control. Metabolite identification was confirmed against reference standards and databases. Metabolites were annotated against the Human Metabolome Database (HMDB) and Kyoto Encyclopedia of Genes and Genomes (KEGG) database.

### Statistical analysis

For conventional parameters, continuous variables were expressed as mean ± standard deviation (SD) and compared using the Student’s *t*-test. Categorical data were presented as frequencies and analyzed using the chi-square test. For ranked or ordinal data, the rank-sum test was applied, with the Wilcoxon rank-sum test utilized for further pairwise comparisons. A P-value of < 0.05 was considered statistically significant.

Data normalization: In brief, LC-MS/MS peak areas were normalized using total-area normalization (sum of all detected metabolite areas per sample), followed by Pareto scaling prior to multivariate analysis. Relative fold changes (Log_2_FC) represent disease group mean peak area relative to healthy control mean peak area (Log_2_[disease/control]).

For metabolomics data processing and analysis, raw LC-MS data were converted using ProteoWizard software and subsequently processed with XCMS software for peak identification, alignment, and integration. The processed data were then imported into MetaboAnalyst 5.0 for univariate and multivariate statistical analyses. Specifically, one-way ANOVA and orthogonal partial least squares discriminant analysis (OPLS-DA) were performed to compare metabolic profiles between HSK-Control and BKC-Control groups. Differential metabolites were screened based on a variable importance in projection (VIP) score > 1 and a P-value < 0.05. Benjamini-Hochberg (BH) false discovery rate (FDR) correction was applied for multiple comparisons across metabolites; metabolites with FDR-corrected p-value <0.05 were retained as statistically significant. OPLS-DA models were validated by 1,000-permutation testing (p < 0.001 for both HSK-Control and BKC-Control models). All differential metabolites with direction of regulation, Log_2_FC, VIP scores, individual raw p-values, and BH FDR-corrected p-values were analyzed. Then a direct OPLS-DA analysis comparing HSK versus BKC by OPLS-DA model with 1,000-permutation testing confirming was used to detect candidates as differential biomarkers for HSK/BKC discrimination. Subsequently, hierarchical cluster analysis, radar chart mapping, metabolic pathway analysis, and regulatory network analysis were conducted based on these differential metabolites.

## Results

### Patient characteristics

A total of 40 patients (43 eyes) were included in this study. The cohort comprised 9 males (22.5%) and 31 females (77.5%), with a mean age of 20.31 ± 10.23 years (range: 3–54 years). Mean intraocular pressure (IOP) was 14.41 ± 3.95 mmHg. Following treatment, all patients demonstrated healed ocular surfaces with negative fluorescein staining, confirming therapeutic efficacy (range: 10–19.4 mmHg). No statistically significant differences were observed in age, sex distribution, or IOP among the three study groups: health control, HSK, and BKC (P > 0.05 for all comparisons) (**Table 1**).

**Table 1:** Population characteristics.

		Controls	HSK	P	BKC	P
Gender (n)	Male	4	6	0.42	4	0.55
	Female	11	18		15	
Age (years)	Range	18-25	3-45	0.546	5-54	0.76
	Mean	21.80 ± 3.23	20.04 ± 12.12		19.35 ± 12.05	
Intraocular Pressure	Range	10-19.4	10.6-18.7	0.590	10.4-19.1	0.28
	Mean	14.5 ± 3.5	14.8 ± 4.4		13.9 ± 3.7	

Control, health volunteers; HSK, herpes simplex keratitis; BKC, blepharokeratoconjunctivitis; P, p value for statistics.

Following treatment, all patients showed healed ocular surfaces with negative fluorescein staining, confirming the effectiveness of the therapeutic interventions.

### Potential tear metabolic biomarkers for HSK *vs*. BKC discrimination

**Figure 1:**
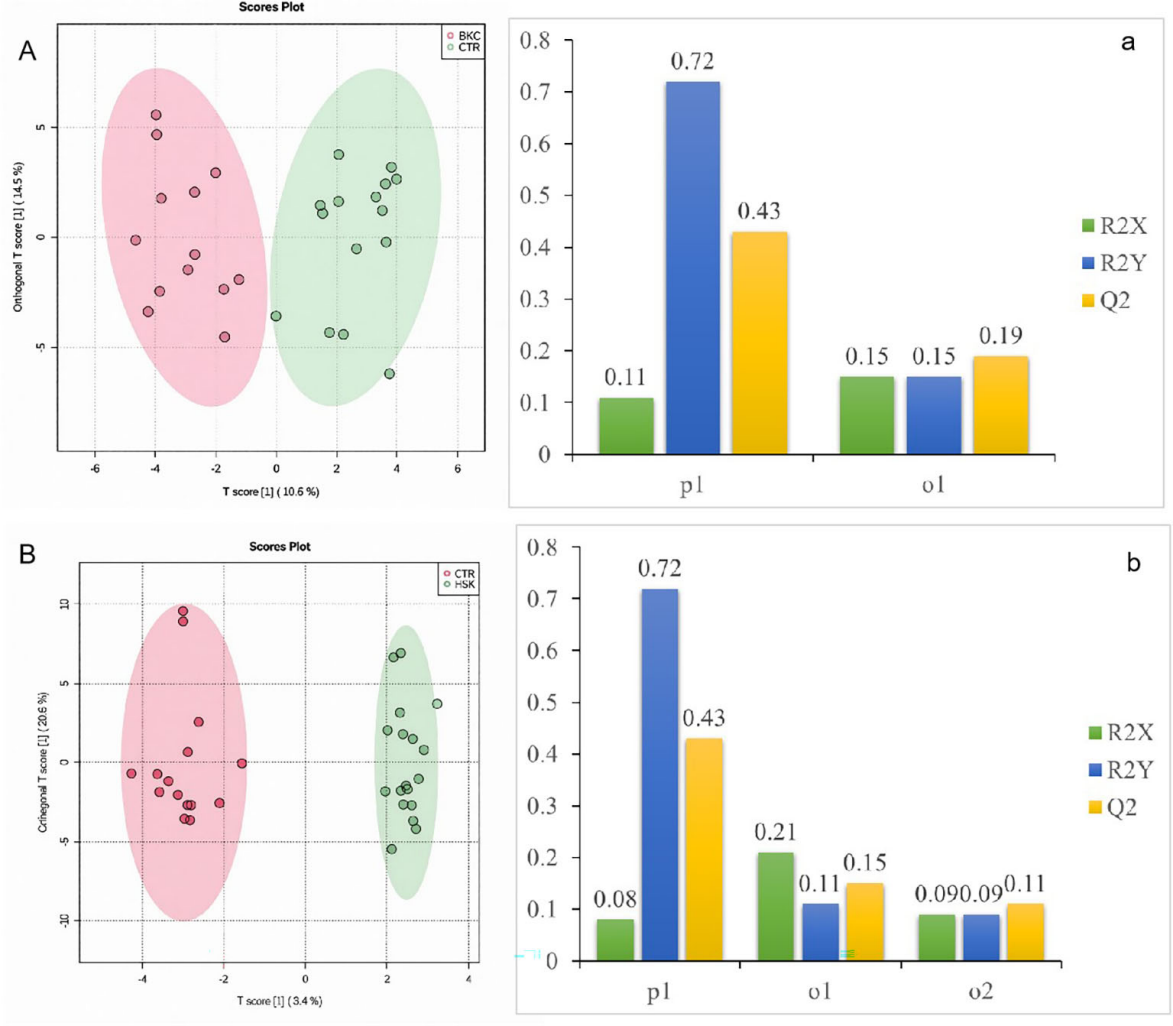
Orthogonal partial least squares-discriminant analysis and model quality testing of metabolic components in HSK-control and BKC-control. HSK, herpes simplex keratitis; BKC, blepharokeratoconjunctivitis; CTR, healthy volunteer. On the X axis, p means first predictive component; o means first orthogonal component. Y axis of a and b means percentage of explained variance. **(A)** The OPLS-DA score plot for HSK-Control shows complete separation between groups, indicating distinct inter-group differences. **(a)** The HSK-Control OPLS-DA model exhibits a high R²Y value, effectively modeling inter-group differences. The acceptable Q² value confirms robust predictive capability of the model. **(B)** The OPLS-DA score plot for BKC-Control demonstrates complete separation between groups, revealing significant inter-group differences. **(b)** The BKC-Control OPLS-DA model shows a high R²Y value, successfully capturing inter-group differences. The acceptable Q² value validates the model’s strong predictive performance.

In the tears, 11 of 171 annotated metabolites were confirmed as valid analytes. A distinct inter-group separation was observed between HSK-Control and BKC-Control groups, indicating significant metabolic differentiation (**Figures 1A, B**). This distinct inter-group separation was validated by model performance metrics (**Figures 1a, b**). Considering the risk of overfitting with high-dimensional metabolomics data due to a relatively limited sample size, 1,000-permutation testing was performed for OPLS-DA models. The permutation tests demonstrate that the observed model performance metrics (R²Y and Q²) are significantly superior to those obtained from randomly permuted data (all p < 0.001), providing strong evidence against overfitting.

Comparative analysis identified 21 differential metabolites in HSK versus controls and 19 in BKC versus controls (without FDR correction). Of these, 13 were common to both conditions, likely reflecting non-specific inflammatory responses. After excluding these shared metabolites and FDR correction, there were 5 metabolites statistically differential between HSK-control and 4 metabolites between BKC-control with high quality of sensitivity and specificity (demonstrated by ROC verification, AUC>0.79). There was no differential metabolite between HSK and BKC (**Table 2**). These candidates and its best combination panel (4-Dodecylbenzenesulfonic Acid+Carnosol+Sorbitol) represent promising tear-based biomarkers for the diagnosis and differential diagnosis of HSK-Control, BKC-Control, and BKC-HSK. Their comparisons and evaluation for diagnostic values were listed in **Table 2**.

**Table 2:** Differential metabolites between BKC-HSK, HSK-control and BKC-control.

Biomarker	VIP	Raw P value	FDR-p value	Log2.Fold.Change	AUC (95% CI)	Sensitivity	Specificity	Optimal cutoff	Comparison
4-Dodecylbenzenesulfonic Acid	3.00	0.00	0.00	-0.78	0.93 (95% CI: 0.83~1.00)	0.83	0.93	6.92	HSK vs. Control
L-Isoleucine	2.39	0.00	0.04	-1.29	0.85 (95% CI: 0.71~0.98)	0.89	0.80	0.27	
Carnosol	1.90	0.00	0.03	-2.06	0.79 (95% CI: 0.62~0.96)	0.89	0.73	0.13	
L-Phenylalanine	2.46	0.00	0.01	2.46	0.86 (95% Cl: 0.73~0.99)	0.89	0.80	0.12	
Pantothenol	3.46	0.00	0.00	3.46	0.96 (95% CI: 0.89~1.00)	0.94	1.00	0.02	
L-Isoleucine	2.98	0.00	0.01	2.98	0.94 (95% Cl: 0.85~1.00)	1.00	0.80	0.24	BKC vs. Control
L-Phenylalanine	2.98	0.00	0.00	2.98	0.94 (95% CI: 0.86~1.00)	1.00	0.80	0.11	
Sorbitol	3.40	0.00	0.01	3.40	1.00 (95% Cl: 1.00~1.00)	1.00	1.00	0.01	
Pantothenol	3.26	0.00	0.02	3.26	0.96 (95% Cl: 0.89~1.00)	0.85	1.00	0.02	
The best combined panel (4-Dodecylbenzenesulfonic Acid+Carnosol+Sorbitol)					0.68 (95% Cl: 0.49~0.87)	0.94	0.46	0.47	BKC VS. HSK

Control, health volunteers; HSK, herpes simplex keratitis; BKC, blepharokeratoconjunctivitis; VIP, variable importance in projection; P, p value for statistics. FDR, false discovery rate; AUC, area under receiver operating characteristic (ROC) curve.

### Metabolomics characteristics of HSK tears

**Figure 2:**
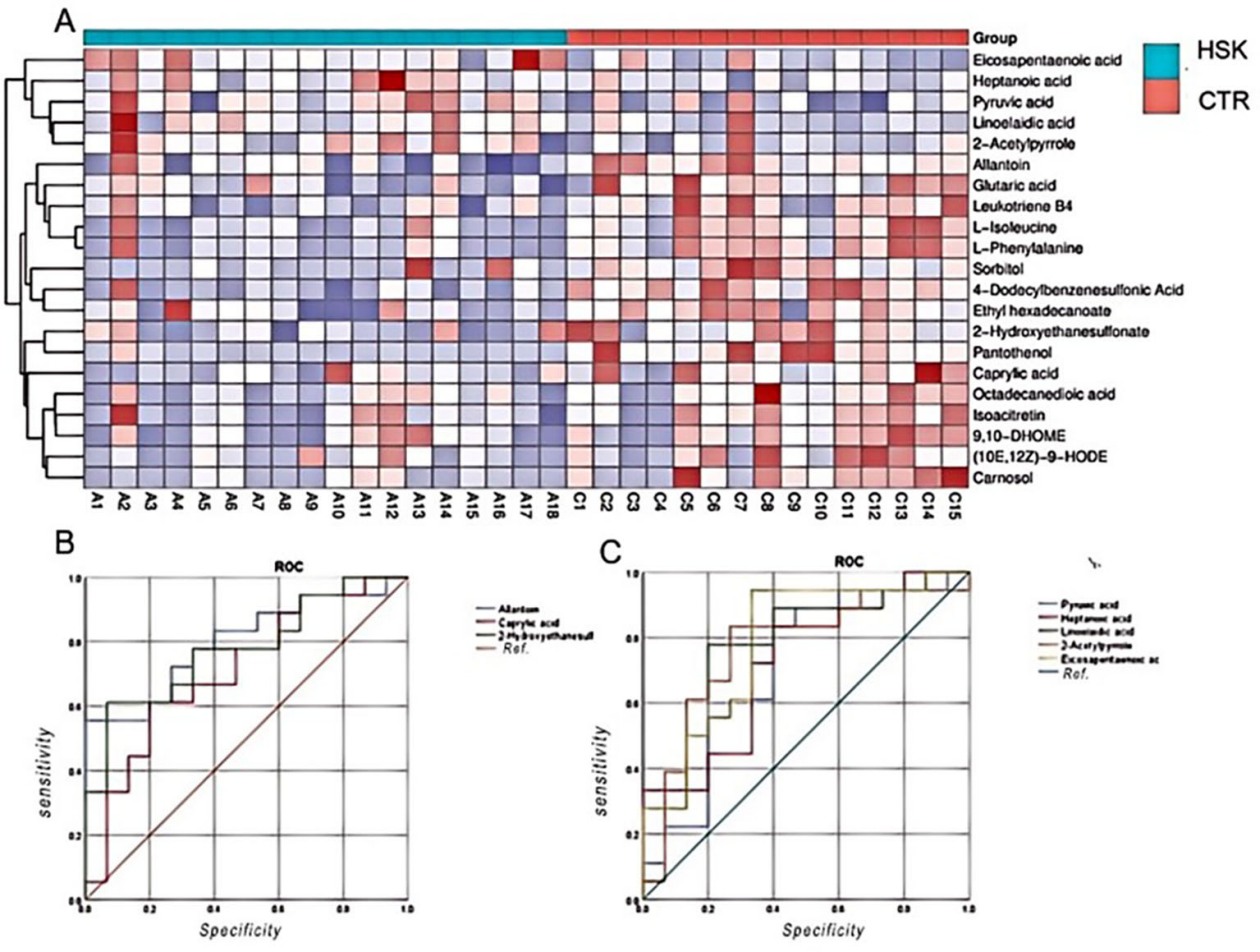
Differential metabolites between HSK and health controls. HSK, herpes simplex keratitis; CTR, healthy volunteer. **(A)** Hierarchical clustering analysis plot derived from HSK-Control group filtered by OPLS-DA VIP ≥1 and p < 0.05; **(B)** ROC curve plot for down-regulated metabolites in HSK-Control group, Allantoin showed the largest area under the curve (AUC); **(C)** ROC curve plot for up-regulated metabolites in HSK-Control group, Pyruvic acid and Caprylic acid showed the largest AUC.

**Figure 3:**
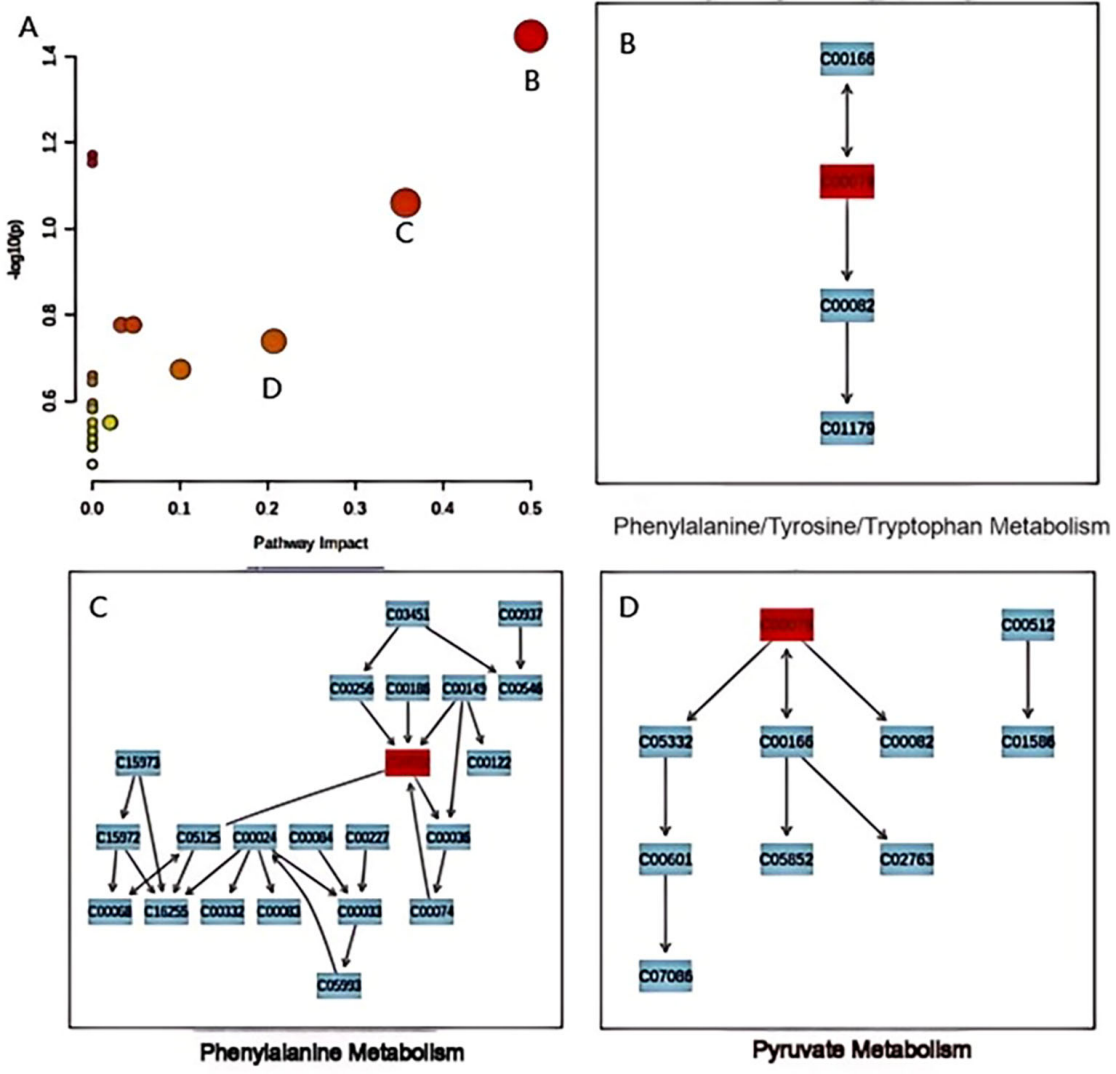
Metabolic pathway analysis for differential marker between HSK-control. HSK, herpes simplex keratitis; CTR, healthy volunteer. **(A)** Metabolic pathway analysis bubble plot, where Y-axis represents the degree of inter-group differences and X-axis represents impact values; **(B)** Biosynthesis and conversion pathway of phenylalanine, tyrosine and tryptophan; **(C)** Phenylalanine metabolic pathway; **(D)** Pyruvate metabolic pathway.

Twenty-one metabolites were significantly altered in HSK patient tears compared to controls without FDR correction (**Figure 2**). Of these 21 metabolites, 5 were upregulated and 16 were downregulated. Metabolic pathway enrichment analysis revealed the most affected pathways were: phenylalanine metabolism, pyruvate metabolism, tricarboxylic acid cycle, and phenylalanine/tyrosine/tryptophan biosynthesis/metabolism. Among them, the phenylalanine-tyrosine-tryptophan pathway demonstrated the highest impact (Impact > 0.5), indicating profound metabolic disruption in HSK tears (**Figure 3**). However, only five metabolites (Dodecylbenzenesulfonic Acid, L-Isoleucine, L-Phenylalanine, Pantothenol, Carnosol) among them represent promising tear-based biomarkers for HSK-control differentiation after FDR correction and receiver operating characteristic (ROC) curve analysis, lower expression in HSK (**Table 2**).

### Metabolomic features in BKC tears

**Figure 4:**
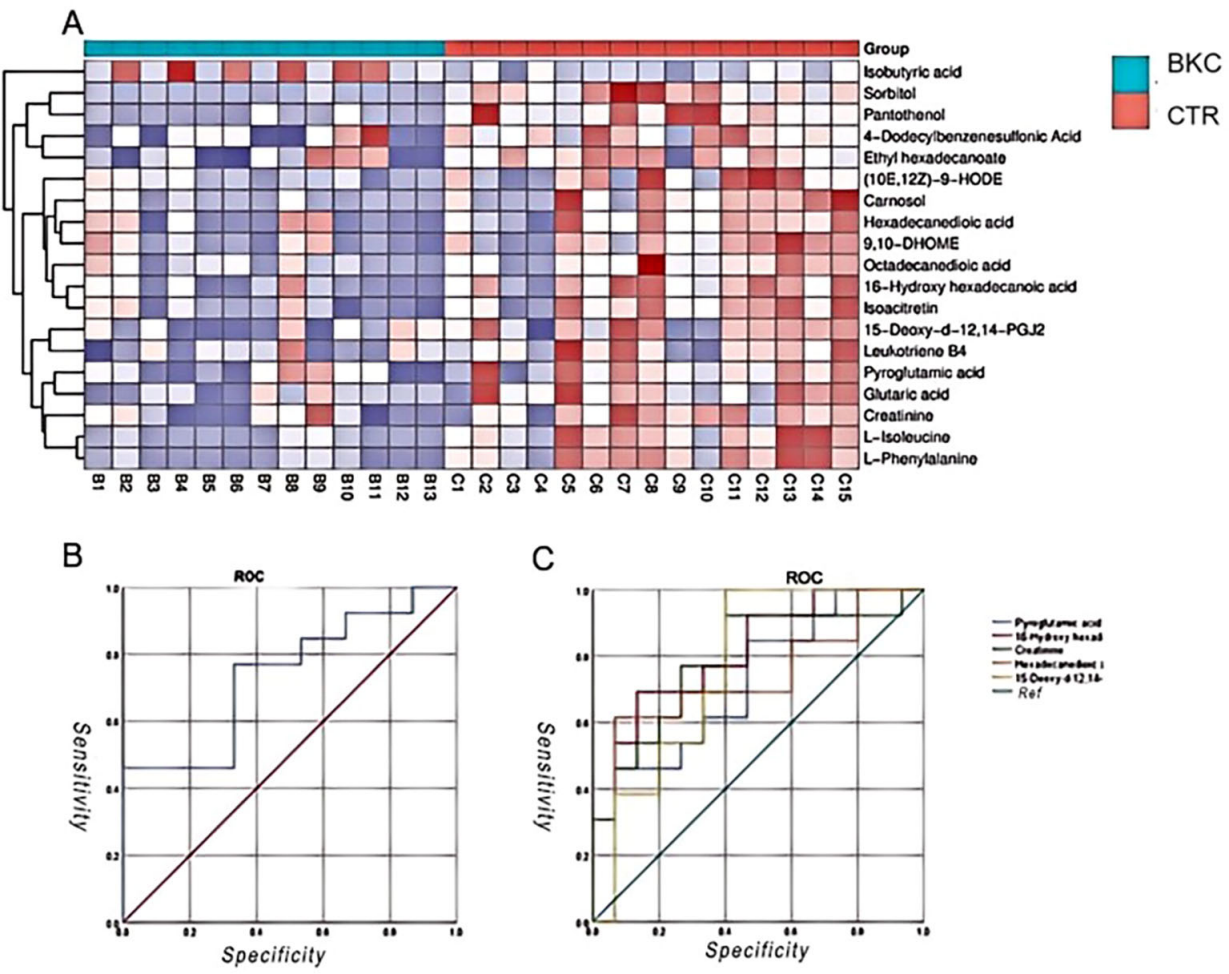
Differential metabolomics between BKC and control. BKC, blepharokeratoconjunctivitis; CTR, healthy volunteer. **(A)** Hierarchical clustering analysis plot derived from BKC-Control group filtered by OPLS-DA VIP ≥1 and p < 0.05; **(B)** ROC curve plot for up-regulated metabolites in BKC-Control group, Isobutyric acid showed the largest area under the curve (AUC); **(C)** ROC curve plot for down-regulated metabolites in BKC-Control group, Creatinine and 16-Hydroxy hexadecanoic acid showed the largest AUC.

The results showed 19 differential metabolites significantly altered in tears from blepharokeratoconjunctivitis (BKC) patients compared to controls (**Figure 4A**). Among these, 1 metabolite was upregulated, while 18 were downregulated. These candidates represent promising tear-based biomarkers for BKC diagnosis (**Figures 4B, C**). However, only five metabolites (L-Isoleucine, L-Phenylalanine, Sorbitol, Pantothenol) among them represent promising tear-based biomarkers for HSK-control differentiation after FDR correction and receiver operating characteristic (ROC) curve analysis, lower expression in HSK (**Table 2**).

## Discussion

Clinical differentiation between BKC and HSK remains challenging in pediatric populations when relying solely on clinical manifestations and slit-lamp biomicroscopy (3, 4). Conventional PCR for ocular HSV-1detection exhibits limitations in detecting low-viral-load samples, particularly in atypical or early-stage infections. In this study, 6 differential metabolites were identified, which could serve as potential tear-based biomarkers for HSK-Control/BKC-Control discrimination. L-isoleucine, L-phenylalanine, pantothenol were all lowered expression in both HSK and BKC. Moreover, 4-dodecylbenzenesulfonic acid, carnosol were lower in HSK, and sorbitol was lower in BKC. A combined panel of metabolites 4-dodecylbenzenesulfonic acid, carnosol and sorbitol showed good specificity and sensitivity for differentiation of BKC/HSK. Meanwhile, the tear metabolomics analysis revealed distinct and consistent metabolic profiles between HSK and BKC cohorts. The treatment outcome further verified the diagnosis along with typical corneal lesion and nPCR results. Considering the above evidence, clinical signs with nPCR result and a metabolite panel (4-dodecylbenzenesulfonic acid+carnosol+sorbitol) may provide a combination for best differentiation of BKC and HSK.

The differential expression patterns of L-isoleucine, L-phenylalanine, and pantothenol reflect a shared inflammatory response, whereas the disease-specific alterations of 4-dodecylbenzenesulfonic acid, carnosol, and sorbitol provide mechanistic insights into HSV-1 infection and chronic immune-mediated ocular surface inflammation, respectively. The combined three-metabolite panel offers a promising, non-invasive adjunct to current diagnostic modalities, with the potential to reduce diagnostic delay and unnecessary antiviral exposure in pediatric patients. The observed changes are discussed in the following in terms of their functional biological consequences for ocular surface homeostasis, including their roles in immunoregulation, energy metabolism and redox regulation, rather than attributing them to a single causal pathway.

The concurrent reduction of L-isoleucine, L-phenylalanine, and pantothenol in both HSK and BKC groups compared to healthy controls suggests a shared metabolic signature of ocular surface inflammation. L-Isoleucine, a branched-chain amino acid (BCAA), has been increasingly recognized for its immunomodulatory properties, including regulation of macrophage polarization and enhancement of antimicrobial peptide production (7). Similarly, L-phenylalanine could diminish M1 macrophage-driven inflammation and modulate effector functions of immune cells through phenylalanine metabolism pathways (8). The observed reduction in phenylalanine levels may therefore indicate active immune cell recruitment and pro-inflammatory polarization at the ocular surface. Notably, decreased phenylalanine has been reported in dry eye disease models and is associated with inflammatory metabolic signatures, supporting its role as a marker of ocular surface inflammation (9). Pantothenol (dexpanthenol, provitamin B5), the alcohol analog of pantothenic acid, is a well-established component of the coenzyme A (CoA) biosynthetic pathway. CoA is an essential cofactor involved in fatty acid synthesis, energy metabolism, and cellular repair processes. The reduction of pantothenol in both disease groups suggests compromised CoA-dependent metabolic capacity at the ocular surface, which may impair corneal epithelial barrier function and delay wound healing (10, 11). Pantothenic acid has been shown to inhibit the growth of intraerythrocytic pathogens, and its depletion may render the ocular surface more susceptible to microbial colonization and persistent inflammation (12). Taken together, the concurrent depletion of these three metabolites represents a non-specific inflammatory response shared between HSK and BKC, reflecting the increased metabolic demand and oxidative stress inherent to ocular surface inflammation in pediatric patients.

In contrast to the shared metabolites, 4-dodecylbenzenesulfonic acid and carnosol were specifically reduced in HSK tears, suggesting a metabolic signature unique to HSV-1 infection. 4-Dodecylbenzenesulfonic acid is a long-chain alkylbenzene sulfonic acid with surfactant properties that belongs to the broader class of tear lipids possessing inherent antimicrobial activity (13). Tear lipids serve as critical components of the ocular surface defense system, providing a hydrophobic barrier against pathogen adhesion and invasion (14). The depletion of this surfactant-like metabolite in HSK may reflect its direct consumption during the host’s antiviral response, or alternatively, may indicate disruption of the tear lipidome caused by HSV-1-induced epithelial damage. Carnosol, a phenolic diterpene derived from rosemary (Rosmarinus officinalis), has attracted considerable attention for its potent anti-inflammatory and antioxidant properties. Mechanistically, carnosol inhibits NLRP3 and NLRC4 inflammasome activation by directly targeting heat-shock protein 90 (HSP90) and suppressing its ATPase activity, thereby blocking caspase-1 activation and subsequent IL-1β and IL-18 maturation (15). Given that HSP90 inhibition has been shown to attenuate HSV-1-induced inflammation (16), the reduced level of carnosol in HSK tears may represent an endogenous anti-inflammatory deficit that fails to adequately counteract HSV-1-triggered inflammasome hyperactivation. Furthermore, carnosol inhibits NF-κB-mediated expression of pro-inflammatory cytokines, including TNF-α and IL-6, during the priming phase of inflammasome activation (15). The depletion of this natural anti-inflammatory mediator in HSK patients may thus contribute to a more severe and sustained inflammatory cascade compared to BKC. These findings collectively suggest that the 4-dodecylbenzenesulfonic acid and carnosol pair may serve as a specific indicator of HSV-1-related ocular surface metabolic disruption.

Sorbitol was specifically reduced in the BKC group, which is of particular pathophysiological significance given the known roles of the polyol pathway in ocular surface homeostasis. Sorbitol, a sugar alcohol generated by aldose reductase-mediated reduction of glucose, is a key intermediate in the polyol pathway. In the context of ocular surface inflammation, the polyol pathway is closely linked to oxidative stress through the consumption of NADPH—a critical cofactor for the regeneration of reduced glutathione (GSH), the primary intracellular antioxidant (17). Paradoxically, the observed reduction of sorbitol in BKC tears may reflect a compensatory downregulation of the polyol pathway to preserve NADPH availability for antioxidant defense. BKC is characterized by chronic inflammation involving meibomian gland dysfunction, lid margin telangiectasia, and recurrent Staphylococcus aureus colonization (18, 19). The associated chronic ocular surface inflammation generates persistent reactive oxygen species (ROS), which may drive adaptive metabolic reprogramming away from sorbitol production toward maintaining redox balance. This is consistent with previous reports demonstrating that aldose reductase and the polyol pathway are dynamically regulated during inflammatory stress (20). Furthermore, sorbitol plays a role in osmotic regulation and cellular hydration homeostasis; its depletion in BKC may exacerbate tear film instability and contribute to the evaporative dry eye component frequently observed in these patients (19). The BKC-specific reduction of sorbitol thus reflects the unique metabolic milieu of chronic, immune-mediated ocular surface inflammation, distinct from the acute, infection-driven metabolic changes in HSK.

The integration of disease-specific metabolites into a combined diagnostic panel represents a promising strategy for improving clinical differentiation between BKC and HSK. The combination of 4-dodecylbenzenesulfonic acid and carnosol (both HSK-specific) with sorbitol (BKC-specific) creates a diagnostic framework that captures both disease-specific and complementary metabolic information, effectively converting the biochemical differences between viral and immune-mediated ocular surface disease into an objective, quantifiable diagnostic metric. Importantly, tear fluid collection is minimally invasive and well-tolerated especially for pediatric populations, making this metabolite panel clinically feasible as a point-of-care adjunct to slit-lamp examination and PCR testing.

However, the combination of clinical signs with nPCR result and a metabolite panel (4-Dodecylbenzenesulfonic Acid+Carnosol+Sorbitol) for HSK/BKC discrimination proposed in this study need further external validation to assess their diagnostic stability, sensitivity, and specificity before clinical translation. Future studies should prioritize independent validation using standardized protocols across diverse populations. Furthermore, the limited sample size prevented correlation of metabolite profiles with specific disease manifestations. Future larger-scale studies should incorporate stratified sampling to address potential heterogeneity and identify metabolite signatures associated with age, gender, disease severity or treatment response.

The analysis employed relative quantification of metabolites due to technical limitations in absolute quantification. Absolute quantification would enable establishment of standardized reference ranges and determination of optimal diagnostic thresholds for metabolite combinations. Such precision could potentially enhance diagnostic accuracy beyond conventional PCR, particularly for atypical or early-stage infections where viral loads are low. A treatment-naive tear collection according to our study protocol is important for avoiding medication-related confounding of the tear metabolome.

This metabolomic analysis represents a single-layer omics approach, without integration with upstream proteomic or microbiome data. A multi-omics framework would elucidate regulatory networks and provide mechanistic insights into the observed metabolic disruptions. Future studies should employ integrated omics strategies to comprehensively map the pathways connecting tear metabolomics with ocular surface inflammation, microbiome dysbiosis, and host immune responses.

In conclusion, this study demonstrates that tear metabolomics can uncover metabolic signatures that effectively discriminate between BKC and HSK, two conditions that frequently pose diagnostic challenges in clinical practice. The combination of clinical signs with nPCR result and a metabolite panel (Carnosol +Sorbitol+4-Dodecylbenzenesulfonic Acid) may be optimal for HSK/BKC discrimination. Validation in prospective, multicenter studies is required to translate these findings into routine clinical practice.

## Data Availability

The original contributions presented in the study are included in the article/supplementary material. Further inquiries can be directed to the corresponding author.
